# A Novel Method for Cancer Subtyping and Risk Prediction Using Consensus Factor Analysis

**DOI:** 10.3389/fonc.2020.01052

**Published:** 2020-06-24

**Authors:** Duc Tran, Hung Nguyen, Uyen Le, George Bebis, Hung N. Luu, Tin Nguyen

**Affiliations:** ^1^Department of Computer Science and Engineering, University of Nevada, Reno, NV, United States; ^2^NTT Hi-Tech Institute, Nguyen Tat Thanh University, Ho Chi Minh City, Vietnam; ^3^Division of Cancer Control and Population Sciences, Hillman Cancer Canter, University of Pittsburgh Medical Center, Pittsburgh, PA, United States; ^4^Department of Epidemiology, University of Pittsburgh Graduate School of Public Health, Pittsburgh, PA, United States

**Keywords:** multi-omics integration, risk score prediction, cancer subtyping, survival analysis, factor analysis

## Abstract

Cancer is an umbrella term that includes a range of disorders, from those that are fast-growing and lethal to indolent lesions with low or delayed potential for progression to death. One critical unmet challenge is that molecular disease subtypes characterized by relevant clinical differences, such as survival, are difficult to differentiate. With the advancement of multi-omics technologies, subtyping methods have shifted toward data integration in order to differentiate among subtypes from a holistic perspective that takes into consideration phenomena at multiple levels. However, these integrative methods are still limited by their statistical assumption and their sensitivity to noise. In addition, they are unable to predict the risk scores of patients using multi-omics data. Here, we present a novel approach named Subtyping via Consensus Factor Analysis (SCFA) that can efficiently remove noisy signals from consistent molecular patterns in order to reliably identify cancer subtypes and accurately predict risk scores of patients. In an extensive analysis of 7,973 samples related to 30 cancers that are available at The Cancer Genome Atlas (TCGA), we demonstrate that SCFA outperforms state-of-the-art approaches in discovering novel subtypes with significantly different survival profiles. We also demonstrate that SCFA is able to predict risk scores that are highly correlated with true patient survival and vital status. More importantly, the accuracy of subtype discovery and risk prediction improves when more data types are integrated into the analysis. The SCFA software and TCGA data packages will be available on Bioconductor.

## 1. Introduction

After 20 years of cancer screening, the chance of a person being diagnosed with prostate or breast cancer has nearly doubled (1–4). However, this has only marginally reduced the number of patients with advanced disease, suggesting that screening has resulted in the substantial harm of excess detection and over-diagnosis. At the same time, 30–50% of patients with non-small cell lung cancer (NSCLC) develop recurrence and die after curative resection (5), suggesting that a subset of patients would have benefited from more aggressive treatments at early stages. Although not routinely recommended as the initial course of treatment, adjuvant and neoadjuvant chemotherapy have been shown to significantly improve the survival of patients with advanced early-stage disease (6–8). The ability to prognosticate outcomes would allow us to manage these diseases better: patients whose cancer is likely to advance quickly or recur would receive the necessary treatment. The important challenge is to discover the molecular subtypes of disease and subgroups of patients (9–12).

Cluster analysis has been a basic tool for subtype discovery using gene expression data. These include hierarchical clustering (HC), neural networks (13–17), mixture model (18–20), matrix factorization (21, 22), and graph-theoretical approaches (23–25). Arguably, the state-of-the-art approach in this area is Consensus Clustering (CC) (26, 27), which is a resampling-based methodology of class discovery and cluster validation (28–30). However, these approaches are not able to combine multiple data types. Although analyses on a single data type could reveal some distinct characteristics for different subtypes, it is not sufficient to explain the mechanism that happens across multiple biological levels.

With the advancement of multi-omics technologies, recent subtyping methods have shifted toward multi-omics data integration. The goal is to differentiate among subtypes from a holistic perspective, that can take into consideration phenomena at various levels (e.g., transcriptomics, proteomics, epigenetics). These methods can be grouped into three categories: simultaneous data decomposition methods, joint statistical models, and similarity-based approaches. Methods in the first category (data decomposition) include md-modules (31), intNMF (32), and LRAcluster (33). These methods assume that there exist molecular patterns that are shared across multiple types of data. Therefore, these methods aim at finding a low dimensional representation of the high-dimensional multi-omics data that retains those patterns. For example, both md-modules and intNMF utilize a joint non-negative matrix factorization to simultaneously factorize the data matrices of multiple data types. In their design, the basis vectors are shared across all data types while the coefficient matrices vary from data type to data type. These two methods, md-modules and intNMF, only differ in the way they iteratively estimate the coefficient matrices. Another method is LRAcluster, which applies the low-rank approximation and singular vector decomposition to generate low dimensional representations of the data and then performs k-means clustering to identify the subtypes. These methods strongly rely on the assumption that all molecular signals can be linearly and simultaneously reconstructed.

Methods in the second category (statistical modeling) include BCC (34), MDI (35), iClusterBayes (36), iClusterPlus (37), and iCluster (38, 39). These methods assume that each data type follows a mixture of distributions and then integrate multiple types of data using a joint statistical model. The parameters of the mixture models are estimated by maximizing the likelihood of observed data. These methods strongly depend on the correctness of their statistical assumptions. Also, due to a large number of parameters and iterations involved, the computation complexity of statistical methods is usually extensive. Therefore, these methods often rely on pre-processing and gene filtering to ease the computational burden.

Methods in the third category (similarity-based) typically construct the pair-wise connectivity between patients (that represents how often the patients are grouped together) for each data type and then integrate multiple data types by fusing the individual connectivity matrices. As these methods perform data integration in the sample space, their computational complexity depends mostly on the number of patients, not the dimensions of features/genes. Therefore, these methods are capable of performing subtyping on a genomic scale. Methods in this category include SNF (40), rMKL-DR (41), NEMO (42), CIMLR (43), and PINS (44, 45). SNF creates a patient-to-patient network by fusing connectivity matrices and then partitions the network using spectral clustering (46). rMKL-DR projects samples into a lower-dimensional subspace and then partitions the patients using k-means. NEMO follows a similar strategy with the difference is that it incorporates only partial data into the integrative analysis. Though powerful, these methods do not account for the noise and unstable nature of quantitative assays. PINS and CIMLR follow two different strategies to address noise and instability. PINS introduces Gaussian noise to the data in order to obtain subtypes that are robust against data perturbation. CIMLR combines multiple gaussian kernels per data type to measure the similarity between each pair of samples. The resulted similarity matrix is then subjected to dimension reduction and k-means to determine the subtypes. Though powerful, the similarity metrics used in these methods (i.e., Gaussian kernel, Euclidean distance) make them susceptible to noise and the “curse of dimensionality” (47) from the high-dimensional multi-omics data.

Here we propose a novel approach, named Subtyping via Consensus Factor Analysis (SCFA), that follows a three-stage hierarchical process to ensure the robustness of the discovered subtypes. First, the method uses an autoencoder to filter out genes with an insignificant contribution in characterizing each patient. Second, it applies a modified factor analysis to generate a collection of factor representations of the high-dimensional multi-omics data. Finally, it utilizes a consensus ensemble to find subtypes that are shared across all factor representations. The software package also includes a model based on Cox regression and Elastic net that is able to predict the risk scores of new patients. In an extensive analysis using 7,973 samples related to 30 different cancer diseases, we demonstrate that our method outperforms other state-of-the-art methods in discovering subtypes with significantly different survival profiles. We also demonstrate that data integration indeed improves the subtyping procedure as subtypes obtained from multi-omics data have more significant Cox *p*-values than subtypes obtained from individual data types. Finally, we demonstrate that the method is able to predict the risk factor of new patients with high accuracy.

## 2. Methods

**Figure 1:**
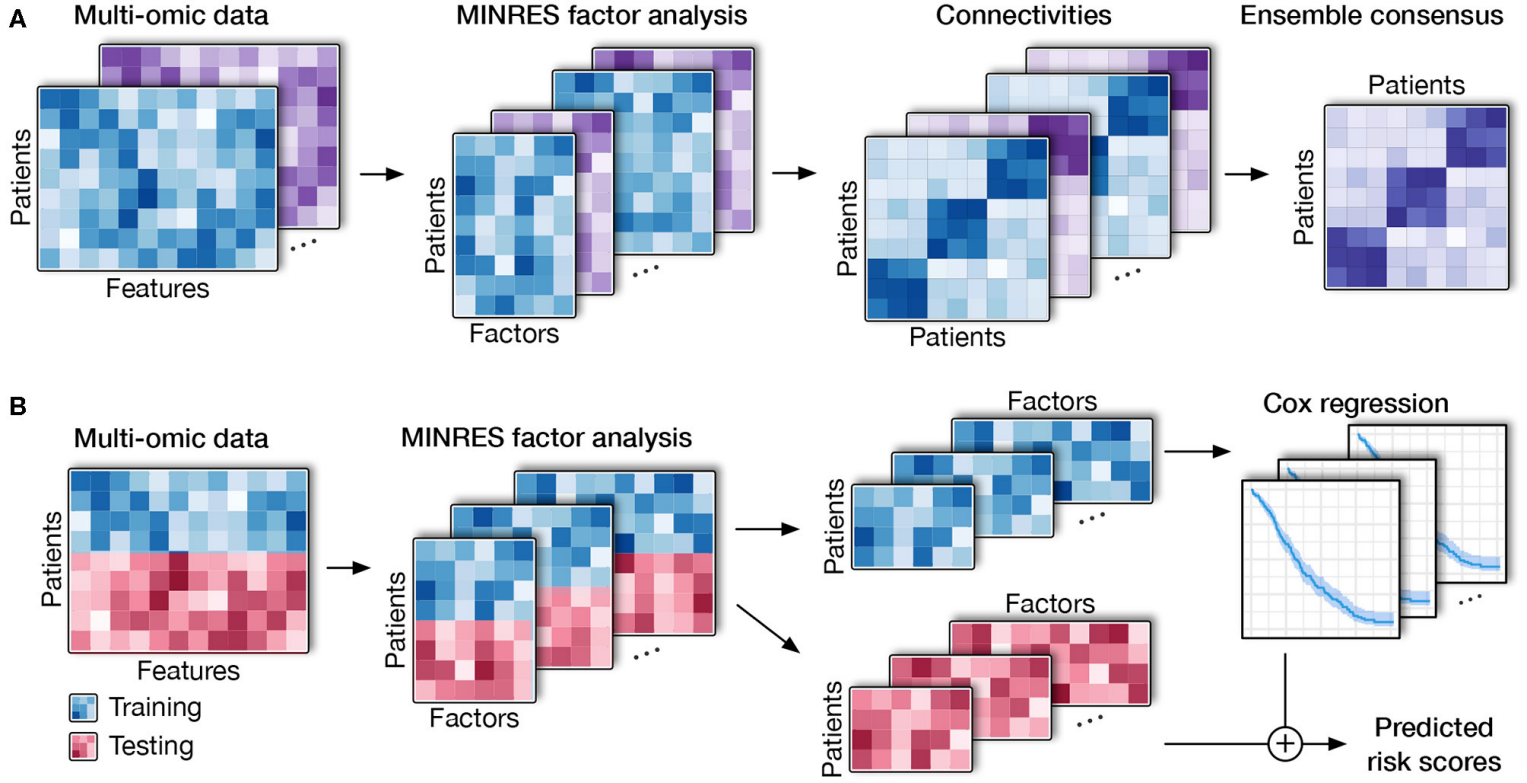
Overall SCFA pipeline. **(A)** Cancer subtyping using multi-omics data. For each of the data matrix, SCFA repeatedly performs factor analysis to generate multiple data representations with different numbers of factors. For each representation, SCFA clusters the data to construct a connectivity matrix. The method next merges all connectivity matrices using an ensemble strategy to obtain the final clustering. **(B)** Risk prediction. SCFA is able to learn from training data (patients with survival information) in order to predict risk scores of patients in testing data (patients without survival information). SCFA first merges training and testing sets together and then performs factor analysis. Using the factor representations of the training set, the method trains a Cox regression model, which will be utilized to predict risk factor of patients in the testing set.

The high-level workflow of SCFA is shown in Figure 1. The framework consists of two main modules: disease subtyping (Figure 1A) and risk assessment (Figure 1B). The input of the subtyping module is a list of data matrices (e.g., mRNA, methylation, miRNA) in which rows represent patients while columns represent genes/features. For each matrix, the method first performs a filtering step using an autoencoder and then repeatedly performs factor analysis (48) to represent the data with different numbers of factors. By representing data with different numbers of factors, we can improve on situations where the projected data do not accurately represent the original data due to noise. Using an ensemble strategy, SCFA combines all of the factor representations to determine the final subtypes.

In the second module, SCFA focuses on predicting the risk scores of patients with unknown survival information. In this module, SCFA combines factor analysis with Cox regression (49, 50) and elastic net (51) to build a prediction model. The method first performs factor analysis on both training (patients with survival information) and testing data (patients without survival information) and then builds a Cox regression model, which can be used to predict the risk scores of patients from the testing data. By default, our software package includes data obtained from The Cancer Genome Atlas (TCGA) that can be used as the training data by default. However, users are free to provide new training data. Using the training data, users can train the model and then predict the risk score of new patients using molecular data.

In the following sections, we will describe in detail the techniques used in the SCFA framework: (i) dimension reduction and factor analysis, (ii) the ensemble strategy for subtyping, and (iii) Cox model and elastic net for risk assessment.

### 2.1. Dimension Reduction and Factor Analysis

Both modules start with dimension reduction and factor analysis. The purpose of dimension reduction is to remove features/genes that play no role in differentiating between patients. This technique was originally introduced in our scDHA method for single-cell analysis (52). Briefly, we utilize a non-negative kernel autoencoder which consists of two components: encoder and decoder. The encoder aims at representing the data in a low dimensional space whereas the decoder tries to reconstruct the original input from the compressed data. By forcing the weights of the network to be non-negative, we capture the positive correlation between the original features and the representative features. Selecting features with high variability in weights would result in a set of features that are informative, non-redundant, and capable of representing the original data.

After the filtering step using the non-negative autoencoder, we perform another dimension reduction step using Factor Analysis (FA) (48). In general, factor analysis aims at minimizing the difference of feature-feature correlation matrix between the latent space and original data. Correlation is a standardized metric, where it takes into account the number of observations and variance of the features during the calculation process. This makes factor analysis robust against scaling and high number of dimensions compared to traditional decomposition such as principle component analysis (PCA), which uses Euclidean distance as the distance metric. To further improve the performance of factor analysis, we adjust the objective of FA to maintain the patient-patient correlation.

Starting with the original correlation matrix, FA finds *k* (number of factors) largest principle components and tries to reproduce the original matrix using those principal components (model matrix). FA iteratively fits the model matrix to the original matrix using optimization algorithms. In our model, we employ the Minimum Residual (MINRES) optimization because it copes better with the small and medium sample size of the input data (53). Also, instead of preserving the relationship between variables, we aim to maintain the overall patient-patient relationships by preserving their Pearson correlations in the representations. By changing the objective, the computational power required is significantly lower as the number of patients (in the scale of hundreds) is much lower than the number of features (in the scale of tens of thousands). Moreover, maintaining the distance between patients in the low dimensional representation would be more beneficial for our desired applications. To avoid overfitting, we repeatedly perform factor analysis with different numbers of factors, resulting in multiple representations of each input matrix. In the clustering module (Figure 1A), all factor representations of all data types (data matrices) are combined using an ensemble strategy to determine the subtypes. In the risk prediction module (Figure 1B), the factor representations of the training data are combined to build the prediction model.

### 2.2. Subtyping Using Consensus Ensemble

Given a collection of factor representations from all data types, we aim at finding patient subgroups that are consistently observed together in all representations (Figure 1A). For each representation, we first determine the optimal number of clusters using two indices: (i) the ratio of *between sum of squares* over the *total sum of squares*, and (ii) the increase of *within sum of squares* when the number of cluster increases (52). After the optimal number of clusters is determined, we use k-means to cluster the underlying factor representation to build a connectivity matrix. To avoid the convergence to a local minimum, we perform k-means clustering using multiple starting points and choose the results with the smallest sum of square error. This process is repeated for all of the representations to obtain a collection of connectivity matrices for all data types.

Finally, we use the Weighted-based meta-clustering algorithm (54) to combine all clustering results from each data representation to determine the final subtyping. In short, the meta-clustering first calculates the weight for each pair of patients regarding their chance to be grouped together. Next, it assigns a weight for each patient by accumulating the weights of all pairs containing this patient. It then computes the weighted cluster-to-cluster similarity from all connectivity matrices. Finally, it partitions the cluster-to-cluster similarity matrix using hierarchical clustering to determine the final subtypes.

### 2.3. Risk Score Prediction

The goal of this module is to calculate the risk score of new patients using their molecular data. This is a supervised learning method that learns from a training set in order to predict the risk scores each patient in the testing set. More specifically, the training set consists of a set of patients with molecular data (e.g., mRNA, methylation, miRNA) and known survival information while the testing set consists of patients with only molecular data. By default, we provide TCGA datasets in our package as training data, but users are free to provide training data if necessary. Using the training data, this module will train the Cox regression model that can be used to predict the risk scores of new patients. Below is the description of the method for one data type and for multi-omics data.

Given a single data type as input, we merge the testing data with training data and then perform dimension reduction and factor analysis to generate multiple representations of this data. For each representation, we use the training data to train the Cox regression model. This model aims at estimating a coefficient β_*i*_ for each corresponding predictor *x*_*i*_ of the input data. After the model is trained, the risk scores for new patients can be calculated as $\exp\left(\overset{n}{\underset{i=1}{\sum }}\beta_{i}x_{i}\right)$, where *n* is the number of features in the factor representation. In the Cox model, the risk score is defined as $\frac{h\left(t\right)}{h_{0}\left(t\right)}$, where *h*(*t*) is the expected hazard at time t, and *h*_0_(*t*) is the baseline hazard when all the predictors are equal zero. Patients with a higher risk score are likely to suffer the event of interest (e.g., vital status or disease recurrence) earlier than the one with a lower risk score. Here we use elastic net (51) implemented in the R-package “glmnet” (55) to fit the model to better cope with the dynamic number of predictors. Elastic net linearly combines Lasso and Ridge penalty during the training process to select only the most relevant predictors that have important effects on the response (the risk scores in this case). We use five-fold cross-validation to select the parameters for the model. The final risk score for each patient is the geometric average of the risk scores resulted from all representations.

In the case of multi-omics data, we repeat the same process (described above) for each data type. We perform factor analysis to produce multiple representations, resulting in a collection of representations from all data types. For a new patient, each representation will produce an estimated risk score. The final risk score for the patient is calculated as the geometric average of all predictions from all representations.

## 3. Result

Here we assess the performance of SCFA using data obtained from 7,973 patients related to 30 different cancer diseases downloaded from The Cancer Genome Atlas (TCGA). For each of the 30 cancer datasets, we downloaded mRNA, miRNA, and methylation data. We also downloaded the clinical data for these patients, which includes vital status and survival information. Using clinical information, we assess the ability of SCFA in both unsupervised subtyping and supervised risk prediction.

### 3.1. Subtypting on 30 TCGA Datasets

Here we compare the performance of SCFA with four state-of-the-art methods: Consensus Clustering (CC) (26, 27), Similarity Network Fusion (SNF) (40), Cancer Integration via Multikernel LeaRning (CIMLR) (43), and iClusterBayes (iCB) (36). CC is a resampling-based approach, while SNF and CIMLR are graph-theoretical approaches. The fourth method, iClusterBayes is a model-based approach and is the enhanced version iClusterPlus. These methods were selected to represent three distinctively different subtyping strategies. Among these methods, CC is the only method that cannot integrate multiple data types. For CC, we concatenate the three data types for the integrative analysis. We demonstrate that SCFA outperforms these methods in identifying subtypes with significantly different survival profiles.

Note that here we focus on unsupervised learning, in which each dataset is partitioned independently without using any external information. For example, when analyzing the glioblastoma multiforme (GBM) dataset, we use only the molecular data (mRNA, miRNA, and methylation) of this dataset to determine the subtypes. For each cancer dataset, we first use each of the five methods (SCFA, CC, SNF, CIMLR, and iClusterBayes) to integrate the molecular data (mRNA, miRNA, and methylation) in order to determine patient subgroups. For each method, we calculate the Cox *p*-value that measures the statistical significance in survival differences between the discovered subtypes. The Cox *p*-values of subtypes discovered by the five methods for the 30 datasets are shown in Table 1. Among the 30 datasets, there are 6 datasets (CHOL, COAD, KIRC, LIHC, OV, and TGCT) for which no method is able to identify subtypes with significant survival differences. In the remaining 24 datasets, SCFA is able to obtain significant Cox *p*-values in all of them while CC, SNF, iClusterBayes, and CIMLR have significant *p*-values in only 8, 12, 11, and 13 datasets, respectively. Also, SCFA has the most significant *p*-values in 19 out of 24 datasets. Regarding time complexity, SCFA, CC, SNF, and CIMLR are able to analyze each dataset in minutes, whereas iClusterBayes can take up to hours to analyze a dataset.

**Table 1:** Cox *p*-values of subtypes identified by SCFA, CC, SNF, iClusterBayes (iCB), and CIMLR for 30 TCGA datasets.

	**SCFA**	**CC**	**SNF**	**iCB**	**CIMLR**
ACC	3.4e-03	5.4e-04	4.3e-05	9.2e-04	3.4e-01
BLCA	7.2e-03	1.1e-01	1.1e-01	5.1e-01	4.7e-01
BRCA	3.2e-04	2.9e-02	1.2e-01	2.7e-02	4.9e-03
CESC	9.4e-03	5.8e-02	5.1e-01	2e-02	1.9e-01
DLBC	4.3e-06	5.1e-01	7.5e-01	2.9e-01	7.4e-01
ESCA	7.3e-05	7.7e-01	3.9e-01	7.9e-01	5.6e-01
GBM	2.3e-03	3.2e-01	2.1e-02	1.1e-01	8.1e-02
GBMLGG	5.8e-14	1.6e-04	4.8e-14	8e-02	6.4e-10
HNSC	4e-02	5e-01	3.7e-01	3.7e-01	4e-01
KICH	2.3e-13	8.7e-01	7e-01	6.9e-01	4.6e-01
KIPAN	1.4e-19	9.3e-08	2.1e-07	1.6e-09	9.8e-05
KIRP	1.7e-03	4.5e-01	5.3e-03	3e-03	1.9e-02
LAML	5.8e-04	3.9e-02	1.7e-03	9e-01	1.4e-04
LGG	6.5e-15	6.6e-07	1.6e-14	1.1e-01	8.3e-15
MESO	1.6e-04	3.1e-01	4.2e-04	3.7e-02	1.1e-02
PAAD	6.9e-04	1.1e-02	7.4e-04	2.3e-03	2e-03
SARC	3.3e-03	2.4e-01	4.4e-02	4.3e-02	5.6e-02
SKCM	1.6e-03	6.3e-01	4.8e-01	8.4e-03	7.4e-05
STES	3.9e-02	2e-01	1.6e-01	4.1e-03	3.4e-02
THCA	7.8e-03	7.9e-01	6.2e-01	7.8e-01	8.6e-03
THYM	8.1e-04	1.5e-01	9.7e-02	9e-03	1.2e-01
UCEC	6.5e-03	8.9e-02	1.8e-02	5.9e-02	4.6e-02
UCS	3.4e-02	1.6e-01	8.6e-01	9.6e-01	3.6e-01
UVM	1.3e-06	6.1e-04	1.7e-04	6.6e-02	5.8e-04
CHOL	3.1e-01	7.9e-02	5.7e-01	9.1e-01	3.4e-01
COAD	4.7e-01	5.8e-01	1.3e-01	2.2e-01	5.6e-01
KIRC	1e-01	8.3e-01	6.9e-01	8.3e-01	9.1e-02
LIHC	3.8e-01	8.8e-01	3.3e-01	9.3e-02	1.9e-01
OV	4.2e-01	6.1e-01	4.4e-01	4.6e-01	5.4e-01
TGCT	3.9e-01	7.4e-01	8.4e-01	7.1e-01	8.4e-01
#Significant	24	8	12	11	13

*The cells highlighted in yellow have Cox p-values smaller than 5%. In each row, cells highlighted in green have the most significant p-value. SCFA outperforms other methods by having significant p-values in most datasets (24 out of 30 datasets)*.

**Figure 2:**
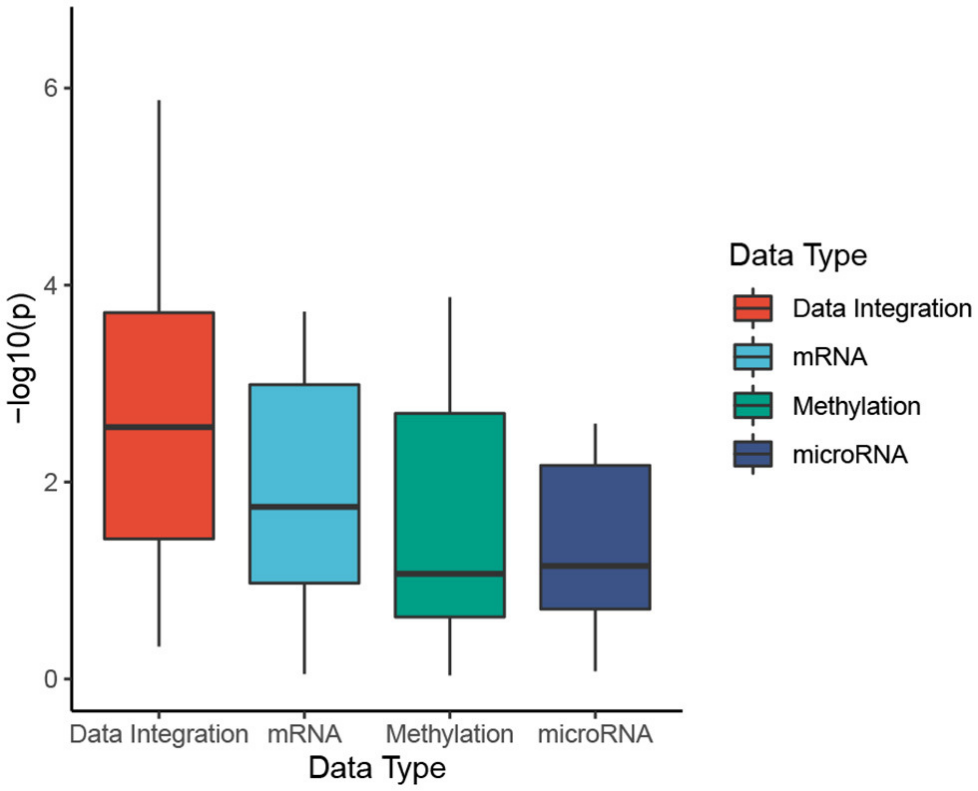
Cox *p*-values of subtypes identified by SCFA. To better understand the usefulness of data integration, we calculate the Cox *p*-values obtained from individual data types and compared them to Cox *p*-values obtained from data integration (when mRNA, miRNA, and methylation are analyzed together). The horizontal axis shows the data types while the vertical axis shows the minus *log*10 *p*-values. Overall the Cox *p*-values obtained from data integration are significantly smaller than those obtained from individual data types (*p* = 0.004 using one-sided Wilcoxon test).

To better understand the usefulness of data integration, we also calculated the Cox *p*-values obtained from individual data types and compared them to Cox *p*-values obtained from data integration (when mRNA, miRNA, and methylation are analyzed together). For each dataset, we perform subtyping using SCFA for each data type and report the Cox *p*-value of the discovered subtypes. The distributions of Cox *p*-values for data integration and for individual data types using SCFA are shown in Figure 2. Among 30 cancer datasets, the Cox *p*-values obtained from data integration has the median −*log*10(*p*) of 2.6, compared to 1.7, 1.1, and 1.1 from gene expression, methylation and miRNA data. Interestingly, subtypes discovered using gene expression data have significantly different survival in 18 over 30 datasets, compared to 10 and 14 of methylation and miRNA data, respectively. The figure also shows that the Cox *p*-values obtained from gene expression data are more significant than those obtained from methylation and miRNA data (*p* = 0.046 using one-sided Wilcoxon test). However, we note that miRNA and methylation also provide valuable information in data integration, when all data types are analyzed together. As shown in Figure 2, the Cox *p*-values obtained from data integration are more significant than those of any individual data type (including mRNA) with a one-sided Wilcoxon test *p*-value of 0.004. This means that each of the three data types provides meaningful contributions to the data integration. To understand how other methods perform with respect to each data type, we also plot the distributions of Cox *p*-values obtained from each data type using CC, SNF, iClusterBayes, and CIMLR (Figure S1). CC is the only method that produces comparable Cox *p*-values across the three data types. SNF and CIMLR perform better using miRNA, while iClusterBayes favors mRNA and miRNA data.

There are four important clinical variables that are available in more than 10 TCGA datasets: age (21 datasets), gender (25 datasets), cancer stages (24 datasets), and tumor grades (12 datasets). To understand the association between these variables and the discovered subtypes, we perform the following analyses: (1) Fisher's exact test to assess the association between gender (male and female) and the discovered subtypes; (2) ANOVA test to assess the age difference between the discovered subtypes; and finally (3) calculate the agreement between the discovered subtypes and known cancer stages/tumor grades using Adjusted Rand Index (ARI) and Normalized Mutual Information (NMI). Figure S2 and Tables S1, S2 show the *p*-values obtained for gender and age. Overall, the four methods, SCFA, CC, SNF, and CIMLR, are not biased toward gender with only some significant *p*-values (Table S1). In contrast, iClusterBayes is subject to gender bias with significant *p*-values in 12 out of 25 datasets (Table S1). The *p*-values of iClusterBayes are significantly smaller than those of other methods (*p* = 0.0007 using one-sided Wilcoxon test). Regarding age, all methods have comparable *p*-values (Table S2). Figure S3 and Table S3 show the ARI values that represent the agreement between the discovered subtypes and known cancer stages and tumor grades. The median ARI of SCFA and SNF are comparable and they are higher than those of CC, iClusterBayes, and CIMLR. Regarding tumor grade, the ARI values of SCFA are higher than the rest. Figure S4 and Table S4 show the NMI values. SCFA has higher NMI values in both comparisons. However, the low NMI and ARI values show that there is a low agreement between the discovered subtypes and known stages/grades.

**Figure 3:**
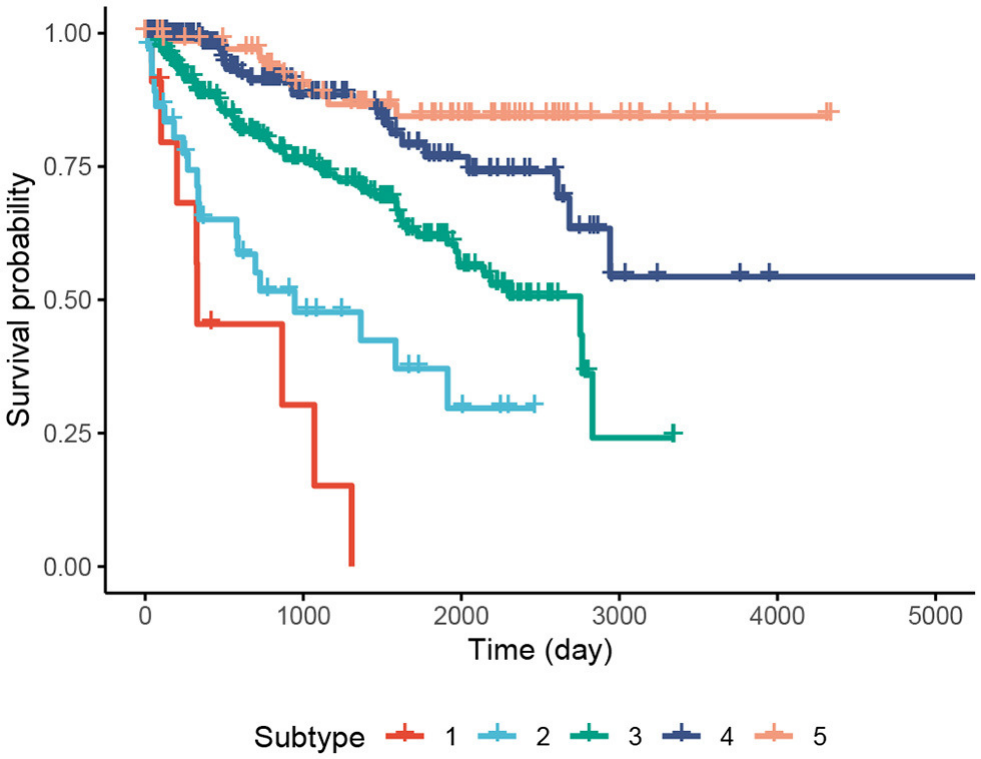
Kaplan–Meier survival analysis of the Pan-kidney (KIPAN) dataset. The horizontal axis represents the time (day) while the vertical axis represents the estimated survival probability.

**Figure 4:**
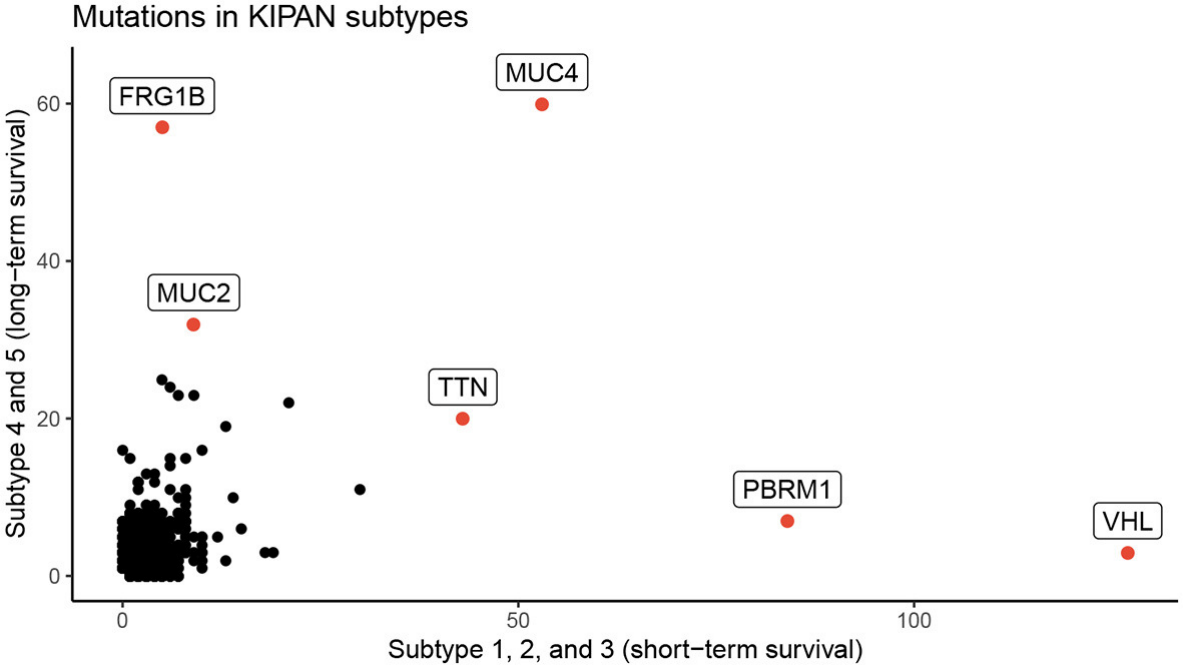
Number of patients in each group for each mutated gene for KIPAN. The horizontal axis represents the count in subtypes with low survival rate (subtype 1, 2, and 3), while the vertical axis shows the count for subtypes with high survival (subtype 4 and 5) rate.

We perform an in-depth analysis for the Pan-kidney (KIPAN) dataset. For this dataset, SCFA discovers five subtypes, each with a very different survival probability (Figure 3). Subtype 1 has the lowest survival probability while Subtype 5 has the highest survival probability. All patients of Subtype 1 die within 3 years whereas 85% of patients in Subtype 5 survive at the end of the study (after 15 years). We also perform variant analysis to look for mutations that are highly abundant in the short-term survival groups (Subtypes 1, 2, and 3) but not in the long-term survival groups (Subtypes 4 and 5), and vice versa. In Figure 4, each point represents a gene and its coordinates represent the number of patients having at least a variant in that gene in each group. In principle, we would look for mutated genes in the top left and the bottom right corners. From this figure, we can identify four notable markers: VHL, PBRM1, MUC4, and FRG1B. Among these, MUC4 has been reported to be associated with exophytic growth of clear cell renal cell carcinoma (56) while VHL linked to a primary oncogenic driver in kidney cancers (57). PBRM1 is also a major clear cell renal cell carcinoma (ccRCC) gene (58). See Supplementary Section 2 and Figures S5–S8 for details.

### 3.2. Risk Score Prediction Using Multi-Omics Data

We also use the same set of data to demonstrate the ability of SCFA in predicting risk score of each patient. For each of the TCGA datasets, we randomly split the data into two equal sets of patients: a training set and a testing set. We use the training set to train the model and then predict the risk for patients in the testing set. The predicted risk scores are then compared with the true vital status and survival information using Cox *p*-value and concordance index (C-index) (59). Concordance index represents the probability that, for a pair of randomly chosen patients, the patient with higher predicted risk will experience death event before the other patient. On the other hand, Cox *p*-value measures how significant the difference in survival when correlating with predicted risk scores. This process is repeated 20 times for each dataset, and the average C-index and −*log*10(*p*) for each dataset are calculated using results from these 20 runs. We note that some datasets do not have enough patients with either event (survive or death), which leads to errors for Cox regression. For that reason, we removed five datasets (DLBC, KIRP, TGCT, THYM, UCEC) from the analysis, and report survival prediction for only 25 datasets without errors.

**Figure 5:**
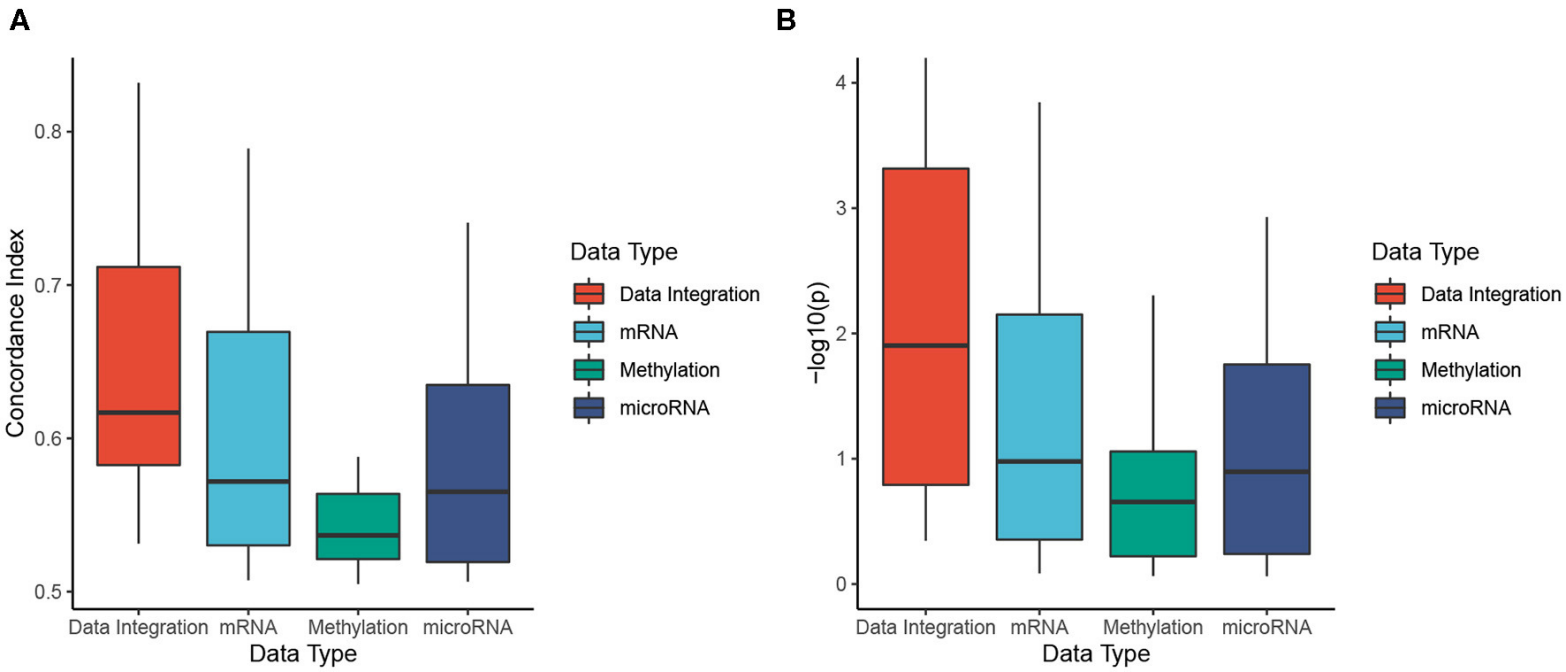
Evaluation of risk prediction using concordance index (C-index) and Cox *p*-values. For each dataset, we calculate the C-index and Cox *p*-values between predicted risk scores and known survival of patients. To better understand the usefulness of data integration, we calculate the C-index and Cox *p*-value obtained from individual data types and compared them to those obtained from data integration. **(A)** Distributions of C-indices for data integration and individual data types. **(B)** Distributions of Cox *p*-values for data integration and individual data types. SCFA is able to predict risk scores that are highly correlated to true survival with a median C-index of 0.62 and Cox *p*-value of 0.01. In addition, the prediction is more accurate when all data types are analyzed together. The C-indices are significantly higher and the *p*-values are significantly smaller when all data types are combined (*p* = 0.0007 and *p* = 0.002 using one-sided Wilcoxon test).

Figure 5 shows the distributions of C-indices and Cox *p*-values (in minus log10 scale), while Table 2 shows the exact values calculated for each dataset. We calculate the C-index and Cox *p*-value obtained from individual data types and compared them to those obtained from data integration (when mRNA, miRNA, and methylation are analyzed together). As shown in Figure 5A, the accuracy of the prediction using data integration is generally higher than the accuracy obtained from individual data types. Predictions using data integration have a median C-index of 0.62, compared to 0.57, 0.54, and 0.57 when using mRNA, methylation, and miRNA, respectively. Similar results are also observed in the evaluation using Cox *p*-values (Figure 5B). The Cox *p*-values obtained from data integration has the median −*log*10(*p*) of 1.9, compared to 1.0, 0.7, and 0.9 for mRNA, methylation, and miRNA. The results demonstrate that we can potentially predict the risk score of each patient using only molecular data. More importantly, the prediction using multi-omics data is generally more accurate than using individual data types.

**Table 2:** Risk score prediction evaluated by concordance index (C-index) and Cox *p*-values.

**Dataset**	**C-index**				**−***log***10(***p***)**			
	**Integration**	**mRNA**	**Methylation**	**microRNA**	**Integration**	**mRNA**	**Methylation**	**microRNA**
ACC	0.78	0.79	0.59	0.72	3.32	3.84	0.66	2.73
BLCA	0.59	0.55	0.55	0.54	2.44	1.1	0.9	0.73
BRCA	0.62	0.55	0.52	0.51	1.38	0.77	0.28	0.14
CESC	0.68	0.63	0.54	0.64	3.42	2.15	1.4	2.02
CHOL	0.56	0.56	0.51	0.55	0.38	0.36	0.2	0.24
COAD	0.56	0.52	0.51	0.57	0.52	0.09	0.09	0.48
ESCA	0.53	0.52	0.5	0.51	0.35	0.09	0.18	0.06
GBM	0.55	0.51	0.53	0.53	2.44	0.3	1.04	1.12
GBMLGG	0.77	0.79	0.72	0.73	14.1	11.56	4.83	5.14
HNSC	0.59	0.59	0.51	0.55	1.41	1.81	0.22	0.48
KICH	0.68	0.6	0.63	0.57	1.35	0.62	2.3	1.31
KIPAN	0.79	0.77	0.73	0.74	24.42	14.53	11.65	20.54
KIRC	0.58	0.59	0.54	0.6	0.79	1.24	0.5	0.94
LAML	0.63	0.61	0.56	0.59	2.45	1.94	1.06	1.16
LGG	0.77	0.78	0.73	0.73	14.02	11.44	5.21	7.53
LIHC	0.62	0.53	0.55	0.57	1.9	0.36	0.86	0.9
MESO	0.72	0.69	0.53	0.63	4.46	3.72	0.22	2.93
OV	0.54	0.51	0.53	0.51	0.41	0.12	0.72	0.14
PAAD	0.71	0.67	0.56	0.59	3.35	2.58	0.79	1.75
SARC	0.62	0.57	0.53	0.53	1.19	0.98	0.19	0.26
SKCM	0.61	0.53	0.53	0.52	2.32	0.55	0.32	0.24
STES	0.54	0.51	0.52	0.51	0.4	0.11	0.29	0.16
THCA	0.66	0.53	0.54	0.51	1.26	0.44	0.33	0.57
UCS	0.58	0.53	0.51	0.51	0.68	0.15	0.06	0.08
UVM	0.83	0.67	0.69	0.72	2.62	1.14	2.87	1.33

## 4. Conclusion

In this article, we presented a novel method (SCFA) for disease subtyping and risk assessment using multi-omics data. The contribution of SCFA is two-fold. First, it utilizes a robust dimension reduction procedure using autoencoder and factor analysis to retain only essential signals. Second, it allows researchers to predict risk scores of patients using multi-omics data—the attribute that is missing in current state-of-the-art subtyping methods.

To evaluate the developed method, we examined data obtained from 7,973 patients related to 30 cancer diseases downloaded from The Cancer Genome Atlas (TCGA). SCFA was compared against four state-of-the-art subtyping methods, CC, SNF, iClusterBayes, and CIMLR. We demonstrate that SCFA outperforms existing approaches in discovering novel subtypes with significantly different survival profiles. We also demonstrate that the method is capable of exploiting complementary signals available in different types of data in order to improve the subtypes. Indeed, the Cox *p*-values obtained from data integration are more significant than those obtained from individual data types.

To further demonstrate the usefulness of the developed method, we also performed a risk assessment using molecular data. We demonstrate that SCFA is able to predict risk scores that are highly correlated with vital status and survival probability. The correlation between predicted risk scores and survival information has a median of 0.62 and can be as high as 0.83. More importantly, we demonstrate that the risk prediction becomes more accurate when more data types are involved.

## Data Availability Statement

TCGA datasets were downloaded from http://firebrowse.org/. The docker contains the environment and scripts used in this article are available at: http://scfa.tinnguyen-lab.com. The current version of SCFA can be found at: https://github.com/duct317/SCFA. The SCFA software and TCGA data package will be available in the next release of Bioconductor.

## Author Contributions

DT and TN conceived of and designed the approach. DT implemented the method in R, performed the data analysis and computational experiments. HN and UL helped with data preparation and some data analysis. DT, HL, GB, and TN wrote the manuscript. All authors reviewed and approved the manuscript.

## Conflict of Interest

The authors declare that the research was conducted in the absence of any commercial or financial relationships that could be construed as a potential conflict of interest.
