# Supplementary material for: A Novel Method for Cancer Subtyping and Risk Prediction Using Consensus Factor Analysis
Source: Front Oncol. 2020 Jun 24;10:1052. doi: 10.3389/fonc.2020.01052 (PMC7344292; doi:10.3389/fonc.2020.01052)
Supplement: Supplementary file 1 [file Data_Sheet_1.pdf]

Supplementary Material: A novel method for cancer subtyping and risk prediction using consensus factor analysis

1 DISCOVERED SUBTYPES AND CLINICAL VARIABLES

Figure S1 shows the distributions of Cox p-values obtained from each data type using SCFA, CC, SNF, iClusterBayes, and CIMLR. We calculate the Cox p-values for individual data types and for data integration (when mRNA, miRNA, and methylation are analyzed together). SCFA discovers subtypes with a more significant survival difference when using gene expression than using miRNA and methylation. In contrast, SNF and CIMLR perform better using microRNA while iClusterBayes favors mRNA and microRNA data. CC is the only method that produces comparable Cox p-values across the three data types.

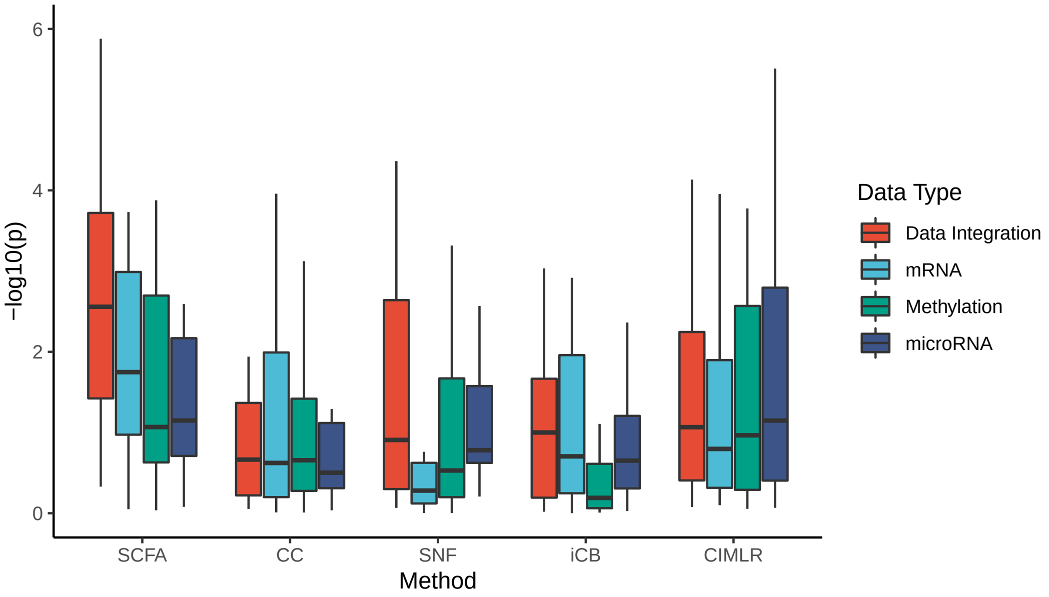

Figure S1. Cox p-values of subtypes identified by SCFA, SNF, iClusterBayes, CC, and CIMLR.

There are four clinical variables that are available in more than 10 datasets: age (21 datasets), gender (25 datasets), cancer stages (24 datasets), and tumor grades (12 datasets). We perform the following analyses: (1) Fisher's exact test to assess the significance of the association between gender (male and female) and the discovered subtypes; (2) ANOVA to assess the age difference between discovered subtypes; and finally (3) calculate the agreement between the discovered subtypes and known cancer stages and tumor grades using Adjusted Rand Index (ARI) and Normalized Mutual Information (NMI).

Briefly, ARI (Hubert and Arabie, 1985) is the corrected-for-chance version of the Rand Index, which measures the agreement between a given clustering and the ground truth. RI is calculated as:

$$RI = \frac{a+b}{a+b+c+d} = \frac{a+b}{\binom{N}{2}} \tag{S1}$$

where a is the number of pairs that belong to the same true group and are clustered together, b is the number of pairs that belong to different true groups and are not clustered together, c is the number of pairs that belong to the same groups and are not clustered together, d is the number of pairs that belong to different groups and are clustered together, and $\binom{N}{2}$ is the number of possible pairs that can be formed from the N patients. The ARI takes values from -1 to 1, with the ARI expected to be 0 for a random subtyping.

Another metric that assesses the agreement between two partitionings is Normalized Mutual Information (NMI), which is a normalized version of Mutual Information (MI). Denoting X as the true labeling of the cells and Y is the partitioning obtained from a clustering method, the NMI is calculated as:

$$NMI = \frac{1}{2} \times \frac{I(X;Y)}{H(X) + H(Y)} \tag{S2}$$

where $I(X;Y)$ is the mutual information between X and Y. $H(X)$ is the entropy of the true partition X and $H(Y)$ is the entropy of the partition obtained from clustering. The NMI value ranges from 0 to 1 in which 1 indicates a perfect match between groups and clusters.

Figure S2 shows the p-value distribution for gender, age, and survival analysis (Cox p-value). Tables S1 and S2 show the p-values obtained for gender and age, respectively. The four methods, SCFA, CC, SNF, and CIMLR, are not biased toward gender with only some significant p-values. In contrast, iClusterBayes is subject to gender bias with significant p-values in 12 out of 25 datasets (Table S1). Regarding age, all methods have comparable p-values (Table S2).

Figure S3 and Table S3 show the ARI values that represent the agreement between the discovered subtypes and known cancer stages and tumor grades. The median ARI of SCFA and SNF are comparable and they are higher than those of CC, iClusterBayes, and CIMLR. Regarding tumor grade, the ARI values of SCFA are higher than the rest. Figure S4 and Table S4 shows the NMI values. SCFA has higher NMI values in both comparisons. However, for both cancer stage and tumor grade, the ARI and NMI values of all methods are low, meaning that there is a low agreement between the known stages/grades and the discovered subtypes using any of the subtyping methods.

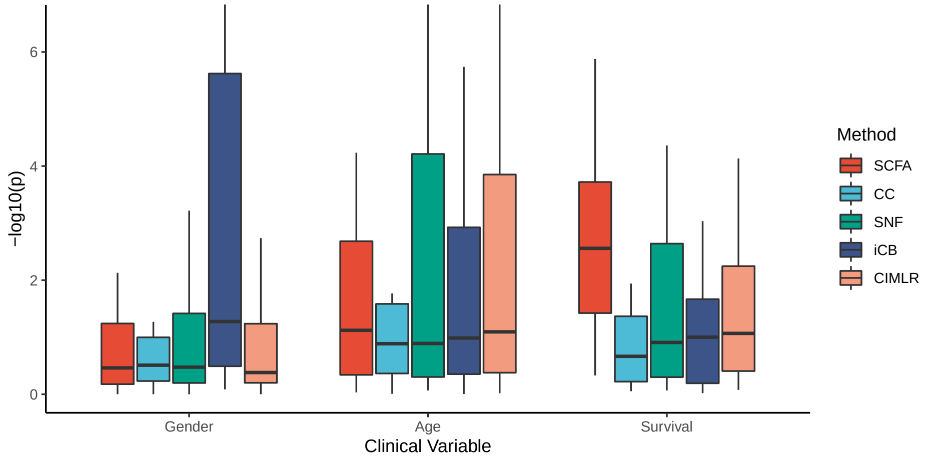

Figure S2. P-values obtained from comparing the discovered subtypes against gender, age, and survival information. Fisher's exact test was used to assess the statistical significance in the association between the discovered subtypes and gender while ANOVA was used to assess age difference. For survival analysis, Cox regression was used to assess the statistical difference in survival profiles. The horizontal axis shows the clinical variables while the vertical axis shows the minus $log10$ p-values.

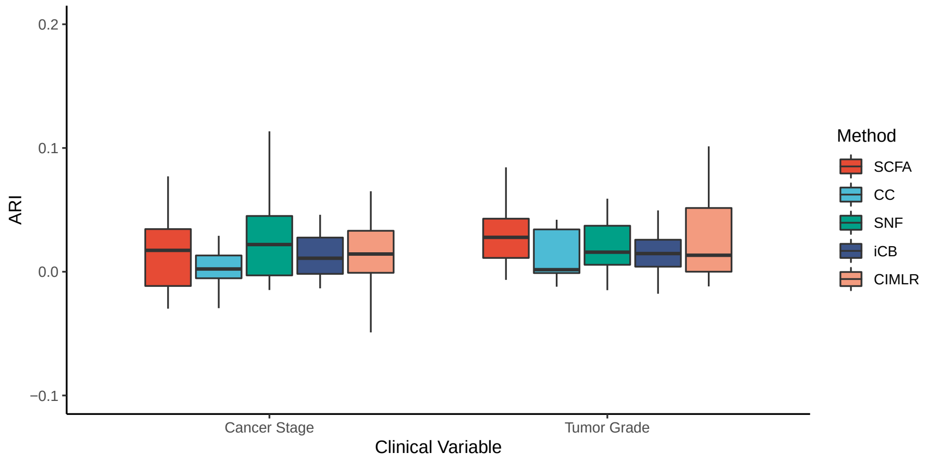

Figure S3. Adjusted Rand Index (ARI) values obtained from comparing the discovered subtypes against known cancer stages (left panel) and tumor grades (right panel).

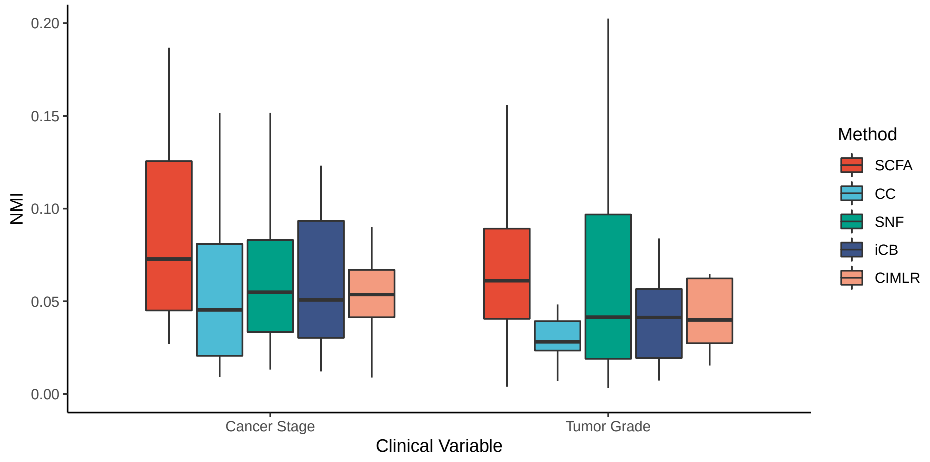

Figure S4. Normalized Mutual Information (NMI) values obtained from comparing the discovered subtypes against known cancer stages (left panel) and tumor grades (right panel).

Table S1. P-values obtained from Fisher's exact test that assesses the statistical significance of the association between the discovered subtypes and gender. NA indicates that there is not enough data to perform the test or all patients have the same gender. Cells highlighted in green have p-values smaller than the significance threshold of 0.05.

	SCFA	CC	SNF	iCB	CIMLR
ACC	4.5e-01	7.1e-01	1.1e-01	3.2e-01	4.9e-01
BLCA	2.4e-01	2.3e-01	2.6e-01	4.4e-01	4.1e-02
BRCA	7.4e-02	5.4e-02	2.1e-01	5.3e-02	9.0e-02
CESC	NA	NA	NA	NA	NA
CHOL	1.0e+00	1.1e-01	3.4e-01	1.6e-01	1.0e+00
COAD	5.7e-01	3.9e-01	7.5e-01	7.6e-45	4.2e-01
DLBC	1.6e-01	5.9e-01	1.0e+00	2.4e-06	3.1e-01
ESCA	8.8e-01	3.0e-01	1.0e+00	6.6e-01	8.3e-01
GBM	6.7e-01	4.6e-01	7.7e-01	8.1e-03	3.6e-01
GBMLGG	5.7e-06	4.9e-01	3.7e-01	6.2e-52	7.5e-01
HNSC	1.9e-01	1.9e-01	9.6e-03	8.2e-01	6.7e-01
KICH	6.5e-01	3.1e-01	2.0e-01	8.2e-03	1.0e+00
KIPAN	7.4e-03	7.0e-04	3.8e-02	7.1e-15	5.8e-02
KIRC	1.4e-02	2.3e-01	2.7e-01	5.8e-01	9.3e-15
KIRP	1.2e-02	1.0e+00	1.1e-03	5.5e-08	7.0e-05
LAML	8.0e-01	7.7e-01	4.3e-01	9.6e-02	6.3e-01
LGG	5.7e-02	1.0e-01	3.6e-01	3.9e-14	4.2e-01
LIHC	5.1e-01	9.0e-04	2.9e-05	3.1e-04	5.7e-06
MESO	2.2e-01	5.3e-01	7.6e-01	2.1e-01	5.8e-02
OV	NA	NA	NA	NA	NA
PAAD	1.0e+00	4.5e-03	6.7e-03	8.1e-01	1.4e-01
SARC	1.6e-05	5.4e-03	2.5e-05	5.6e-12	1.2e-03
SKCM	3.5e-01	6.7e-01	4.2e-01	1.2e-01	6.4e-01
STES	1.3e-02	5.9e-02	6.0e-04	9.9e-05	1.8e-03
TGCT	NA	NA	NA	NA	NA
THCA	8.7e-01	7.9e-01	3.7e-01	4.8e-01	4.6e-01
THYM	6.8e-01	5.7e-01	6.3e-01	5.5e-06	5.3e-01
UCEC	NA	NA	NA	NA	NA
UCS	NA	NA	NA	NA	NA
UVM	5.0e-01	1.0e+00	1.0e+00	6.2e-02	5.2e-01

Table S2. P-values obtained from ANOVA that assesses statistical significance in age difference between the discovered subtypes. Cells highlighted in green have p-values smaller than the significance threshold of 0.05.

	SCFA	CC	SNF	iCB	CIMLR
ACC	NA	NA	NA	NA	NA
BLCA	2.1e-03	1.5e-01	6.6e-03	4.6e-03	2.5e-02
BRCA	1.3e-02	6.2e-02	2.0e-01	6.7e-05	1.4e-04
CESC	4.6e-01	4.0e-02	3.8e-01	1.2e-07	1.3e-01
CHOL	NA	NA	NA	NA	NA
COAD	9.3e-01	4.3e-01	5.4e-01	6.4e-02	3.1e-01
DLBC	8.0e-01	2.4e-01	8.6e-01	4.9e-01	8.3e-01
ESCA	NA	NA	NA	NA	NA
GBM	5.8e-05	3.1e-02	1.4e-02	2.0e-05	2.9e-02
GBMLGG	9.5e-13	1.7e-02	1.2e-17	1.1e-01	2.8e-16
HNSC	1.5e-01	1.1e-01	5.3e-01	9.2e-01	4.2e-01
KICH	3.2e-01	1.3e-01	3.0e-01	4.4e-01	8.1e-02
KIPAN	3.0e-08	1.3e-06	1.2e-08	1.3e-01	8.8e-08
KIRC	1.9e-01	6.8e-01	6.1e-01	9.9e-01	6.4e-01
KIRP	3.7e-03	2.9e-01	2.3e-01	1.0e-01	9.6e-01
LAML	6.9e-03	2.4e-06	6.1e-05	7.9e-02	5.2e-06
LGG	4.3e-11	3.8e-04	1.9e-18	3.4e-01	3.9e-16
LIHC	6.4e-01	2.1e-05	3.3e-05	9.3e-04	1.9e-03
MESO	NA	NA	NA	NA	NA
OV	1.9e-02	4.7e-01	1.3e-01	1.8e-06	2.1e-01
PAAD	7.6e-02	9.8e-01	5.0e-01	1.7e-01	5.5e-01
SARC	NA	NA	NA	NA	NA
SKCM	1.5e-01	8.8e-01	6.1e-03	1.2e-03	1.1e-01
STES	6.1e-01	2.6e-02	5.1e-01	4.5e-01	8.8e-01
TGCT	NA	NA	NA	NA	NA
THCA	5.8e-01	2.7e-01	9.5e-02	6.0e-01	1.3e-02
THYM	NA	NA	NA	NA	NA
UCEC	1.6e-03	6.0e-01	1.3e-07	8.4e-03	1.4e-04
UCS	NA	NA	NA	NA	NA
UVM	NA	NA	NA	NA	NA

Table S3. Adjusted Rand Index (ARI) values obtained from comparing the discovered subtypes against known cancer stages and tumor grades.

	Cancer Stage					Tumor Grade				
	SCFA	CC	SNF	iCB	CIMLR	SCFA	CC	SNF	iCB	CIMLR
ACC	0.05	0.02	0.07	0.04	0.02	NA	NA	NA	NA	NA
BLCA	0.02	0	0.03	0.03	0.02	0.03	-0.01	0.04	0.01	0.02
BRCA	-0.02	0	-0.01	0	0.01	NA	NA	NA	NA	NA
CESC	0	0.01	-0.01	0	0.02	0	0	0.01	0.03	0
CHOL	-0.02	-0.02	-0.01	0	-0.02	NA	NA	NA	NA	NA
COAD	-0.03	-0.01	0	-0.01	0	NA	NA	NA	NA	NA
DLBC	-0.02	0.01	0	0.05	-0.05	NA	NA	NA	NA	NA
ESCA	0.08	0.08	0.07	0	0.07	NA	NA	NA	NA	NA
GBM	NA	NA	NA	NA	NA	NA	NA	NA	NA	NA
GBMLGG	NA	NA	NA	NA	NA	0.03	0.04	0.04	0.01	0.05
HNSC	-0.03	0	-0.01	0	0	-0.01	0	0.01	0	0
KICH	0.05	0	0.11	0.05	0.04	NA	NA	NA	NA	NA
KIPAN	0.07	0.01	0.04	0.03	0.06	0.04	0	0.01	0.01	0.02
KIRC	0.02	-0.03	-0.01	-0.01	-0.01	0.06	0.02	0.02	0.05	-0.01
KIRP	0.03	0.01	0.15	0.02	0.1	NA	NA	NA	NA	NA
LAML	NA	NA	NA	NA	NA	NA	NA	NA	NA	NA
LGG	NA	NA	NA	NA	NA	0.03	0.03	0.04	0.02	0.05
LIHC	0	0	0	0.02	0.03	0.02	0	0.01	0.03	0.01
MESO	-0.01	-0.01	0.03	0	-0.02	NA	NA	NA	NA	NA
OV	0	0.02	0	-0.01	0.01	0.02	0	-0.01	-0.02	0.01
PAAD	0.1	-0.01	0.04	0.12	0.05	0.06	-0.01	0.06	0.06	0.05
SARC	NA	NA	NA	NA	NA	NA	NA	NA	NA	NA
SKCM	0.02	0	0.02	0.01	0.01	NA	NA	NA	NA	NA
STES	0.01	0.02	0.01	0.01	0.01	0	0.04	0	-0.02	-0.01
TGCT	0.03	0.05	0.03	0.02	0.03	NA	NA	NA	NA	NA
THCA	-0.01	0.01	0.01	0.01	0.02	NA	NA	NA	NA	NA
THYM	NA	NA	NA	NA	NA	NA	NA	NA	NA	NA
UCEC	0.01	-0.03	0.04	0.01	0.01	0.08	0.12	0.06	0.02	0.1
UCS	0.03	0	0.05	0.02	-0.03	NA	NA	NA	NA	NA
UVM	0.04	0.03	0.05	0.09	0.03	NA	NA	NA	NA	NA

Table S4. Normalized Mutual Information (MNI) values obtained from comparing the discovered subtypes against known cancer stages and tumor grades.

	Cancer Stage					Tumor Grade				
	SCFA	CC	SNF	iCB	CIMLR	SCFA	CC	SNF	iCB	CIMLR
ACC	0.12	0.06	0.1	0.11	0.06	NA	NA	NA	NA	NA
BLCA	0.05	0.02	0.03	0.04	0.03	0.06	0.05	0.1	0.06	0.05
BRCA	0.03	0.01	0.02	0.02	0.04	NA	NA	NA	NA	NA
CESC	0.04	0.06	0.03	0.05	0.06	0.03	0.01	0.01	0.04	0.04
CHOL	0.15	0.13	0.08	0.2	0.16	NA	NA	NA	NA	NA
COAD	0.08	0.05	0.06	0.05	0.06	NA	NA	NA	NA	NA
DLBC	0.11	0.09	0.07	0.1	0.02	NA	NA	NA	NA	NA
ESCA	0.13	0.12	0.09	0.09	0.09	NA	NA	NA	NA	NA
GBM	NA	NA	NA	NA	NA	NA	NA	NA	NA	NA
GBMLGG	NA	NA	NA	NA	NA	0.06	0.03	0.06	0.01	0.06
HNSC	0.05	0.02	0.01	0.03	0.05	0.06	0.04	0.02	0.03	0.03
KICH	0.19	0.12	0.1	0.12	0.04	NA	NA	NA	NA	NA
KIPAN	0.07	0.05	0.06	0.03	0.05	0.04	0.03	0.02	0.06	0.02
KIRC	0.04	0.07	0.04	0.01	0.01	0.12	0.11	0.1	0.08	0.04
KIRP	0.08	0.02	0.1	0.02	0.07	NA	NA	NA	NA	NA
LAML	NA	NA	NA	NA	NA	NA	NA	NA	NA	NA
LGG	NA	NA	NA	NA	NA	0.09	0.03	0.06	0.01	0.06
LIHC	0.03	0.02	0.02	0.03	0.03	0.01	0.01	0.01	0.04	0.03
MESO	0.09	0.04	0.02	0.05	0.06	NA	NA	NA	NA	NA
OV	0.05	0.02	0.05	0.02	0.04	0.05	0.03	0.02	0.02	0.02
PAAD	0.13	0.04	0.08	0.11	0.06	0.09	0.03	0.1	0.08	0.06
SARC	NA	NA	NA	NA	NA	NA	NA	NA	NA	NA
SKCM	0.04	0.02	0.03	0.04	0.05	NA	NA	NA	NA	NA
STES	0.06	0.05	0.05	0.05	0.05	0	0.02	0	0.01	0.02
TGCT	0.12	0.12	0.09	0.09	0.08	NA	NA	NA	NA	NA
THCA	0.03	0.03	0.03	0.03	0.05	NA	NA	NA	NA	NA
THYM	NA	NA	NA	NA	NA	NA	NA	NA	NA	NA
UCEC	0.05	0.04	0.04	0.07	0.07	0.16	0.07	0.2	0.06	0.15
UCS	0.29	0.15	0.15	0.21	0.11	NA	NA	NA	NA	NA
UVM	0.13	0.08	0.08	0.08	0.11	NA	NA	NA	NA	NA

2 ANALYSIS OF PAN-KIDNEY (KIPAN) DATASET

Figure S5 shows the Kaplan-Meier survival analysis (Kaplan and Meier, 1958) of the discovered subtypes using the KIPAN dataset. SCFA discovers five subtypes, each with a very different survival probability. Subtype 1 has the lowest survival rate while Subtype 5 has the highest survival rate. All patients of Subtype 1 die within three years whereas 85% of patients in Subtype 5 survive at the end of the study (after 15 years). Figure S6 shows the age distribution of each subtype, in which patients in Subtype 1 (low survival) are slightly older than patients in Subtype 5 (high survival) but there is no significant difference in age between the two groups. Patients in Subtypes 2, 3, and 4 are older than those of Subtype 1 (low survival) but they have higher survival probability.

To show the molecular signature of each subtype, we also plot the heatmaps that visualize different subtypes of KIPAN patients on important genes/features. For each data type, we calculate the p-value for each feature using ANOVA and then choose 20 features/genes with the most significant p-value. Figure S6 shows the heatmap for mRNA (left panel), methylation (middle panel) and miRNA (right panel). The methylation data clearly differentiates Subtype 5 (highest survival probability) from the rest. In the listed probes (DNA regions), Subtype 5 has a consistently low level of methylation compared to other subtypes. However, methylation data alone cannot differentiate among the rest of the patients (Subtypes 1, 2, 3, and 4). Using information from mRNA and miRNA, SCFA can further divide the rest of the patients into four subtypes with very different survival profiles.

We also perform variant analysis to look for mutations that are highly abundant in the short-term survival groups but not in the long-term survival groups, as shown in Figure S8. In this figure, each point represents a gene and its coordinates are the number of patients having at least a variant in that gene in each group. In principle, we would look for mutated genes in the top left and the bottom right corners. From this figure, we can identify four notable markers: VHL, PBRM1, MUC4, and FRG1B. Among these, MUC4 is known to be associated with exophytic growth of clear cell renal cell carcinoma (Shinagare et al., 2015). VHL has been reported to be linked to a primary oncogenic driver in kidney cancers (Thomas et al., 2006). PBRM1 is also a major clear cell renal cell carcinoma (ccRCC) gene (Varela et al., 2011).

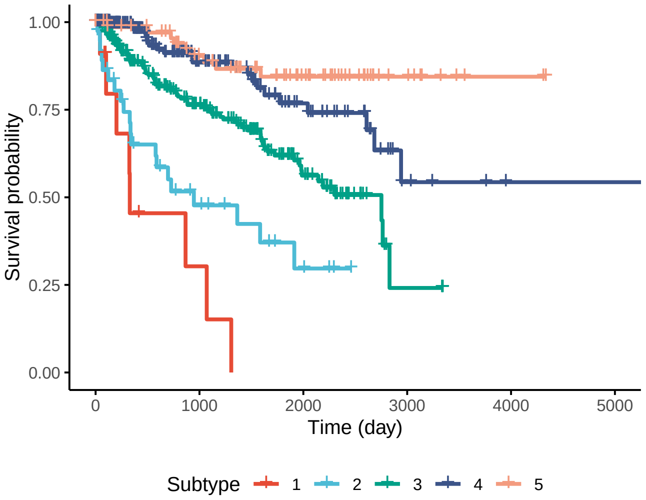

Figure S5. Kaplan-Meier survival analysis of the Pan-kidney (KIPAN) dataset. The horizontal axis represents the time (day) while the vertical axis represents the estimated survival probability.

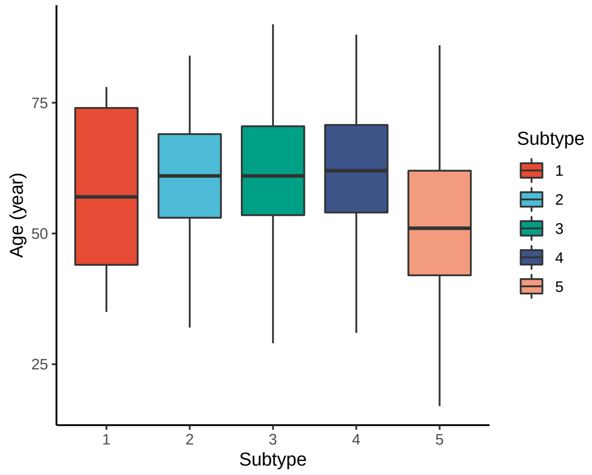

Figure S6. Age distribution for each subtype of the KIPAN dataset.

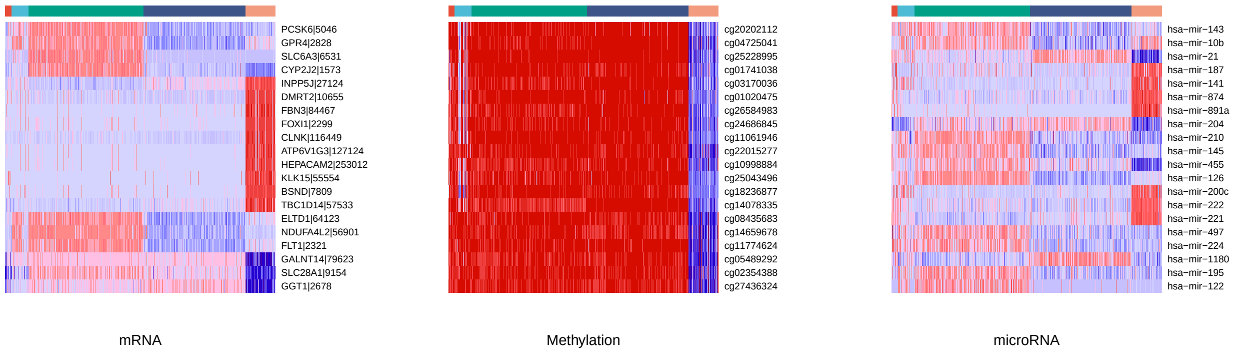

Figure S7. Heatmap of subtypes discovered by SCFA for the KIPAN dataset.

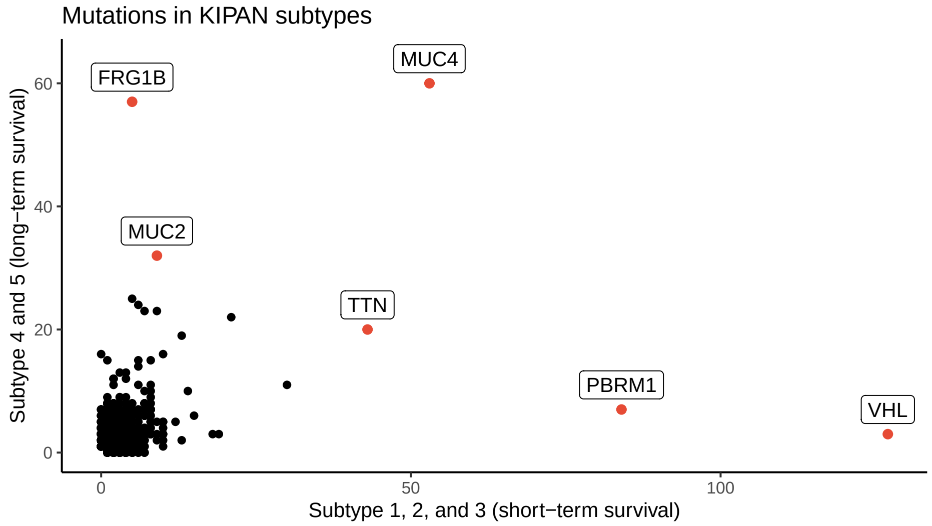

Figure S8. Number of patients in each group for each mutated gene for KIPAN. The horizontal axis represents the count in subtypes with low survival rate (subtype 1, 2, and 3), while the vertical axis shows the count for subtypes with high survival (subtype 4 and 5) rate.

REFERENCES

Hubert, L. and Arabie, P. (1985). Comparing partitions. *Journal of Classification* 2, 193–218

Kaplan, E. L. and Meier, P. (1958). Nonparametric estimation from incomplete observations. *Journal of the American Statistical Association* 53, 457–481

Shinagare, A. B., Vikram, R., Jaffe, C., Akin, O., Kirby, J., Huang, E., et al. (2015). Radiogenomics of clear cell renal cell carcinoma: preliminary findings of the cancer genome atlas–renal cell carcinoma (tcga–rcc) imaging research group. *Abdominal imaging* 40, 1684–1692

Thomas, G. V., Tran, C., Mellinghoff, I. K., Welsbie, D. S., Chan, E., Fueger, B., et al. (2006). Hypoxia-inducible factor determines sensitivity to inhibitors of mtor in kidney cancer. *Nature medicine* 12, 122–127

Varela, I., Tarpey, P., Raine, K., Huang, D., Ong, C. K., Stephens, P., et al. (2011). Exome sequencing identifies frequent mutation of the swi/snf complex gene pbrm1 in renal carcinoma. *Nature* 469, 539–542
